# Enhanced Optical Confinement Enriching the Power Conversion Efficiency of Integrated 3D Grating Organic Solar Cell

**DOI:** 10.3390/polym14204294

**Published:** 2022-10-12

**Authors:** Moshe Zohar, Roy Avrahamy, Shlomo Hava, Benny Milgrom, Evyatar Rimon

**Affiliations:** 1 Electrical and Electronics Engineering Department, Shamoon College of Engineering, P.O. Box 950, Beer Sheva 8410802, Israel; 2 School of Electrical and Computer Engineering, Ben-Gurion University of the Negev, P.O. Box 653, Beer-Sheva 8410501, Israel; 3 School of Electrical Engineering, Jerusalem College of Technology, P.O. Box 16031, Jerusalem 9372115, Israel

**Keywords:** organic solar cells (OSCs), solar energy, photovoltaic (PV), grating structures, light confinement

## Abstract

In this paper, we examine the impact of three-dimensional grating layers embedded at selected locations in an organic solar cell structure to obtain enhanced efficiency. The design, simulations, and optimizations were carried out using an in-house tool based on the rigorous coupled-wave analysis (RCWA) method developed on the MATLAB R2019a platform. An optimal organic solar cell structure design with a top grating layer exhibited an increase of 7.47% in the short-circuit current density compared to an organic solar cell structure with a smooth top layer. The power conversion efficiency (PCE) increase was mainly due to increased light confinement in the thin absorbing layer. Adding an embedded grating layer in the absorption layer resulted in a significant increase in the absorptance spectral bandwidth, where the short-circuit current density increased by 10.88%. In addition, the grating cells yielded a substantial improvement in the cell’s conical absorptance since the existence of a surface plasmon polariton (SPP) in the back metal gratings increases the confinement properties. Further, the effect of a pyramid-shaped embedded grating array was a slight improvement in the PCE compared to the rectangular-shaped grating arrays. We showed that a pyramid-grating can act as a nano black-body layer, increasing the absorption for a wide range of azimuthal and polar incident angles.

## 1. Introduction

Organic solar cells (OSCs) display the desirable features of low weight, semitransparent, non-toxic, low-cost and simple manufacturing processes, and flexibility [1,2,3]. However, their main disadvantage is their low power conversion efficiency (PCE), reaching a record high of 15.6% compared to 26.6% with silicon technology [4]. The primary reasons for this are:

- The narrow absorption band;
- The low mobility of the charge carriers;
- The short diffusion length of the excitons within the organic absorber semiconductor blend.

The last of the above imposes constraints on the absorption layer thickness, which must be thin, with a typical thickness of around 100 to 300 nm [5,6]. For absorber layer thicknesses above this upper value, generated charge carriers will undergo recombination before reaching the contacts, leading to an insignificant amount of photon conversion [5]. Therefore, it is necessary to design non-conventional solar cell structures to increase the absorption and thus obtain a higher short-circuit current density and a higher PCE. While this study focuses on the geometrical-structural properties of the polymer solar cell, we also note here the rising interest in the employment of deep learning for screening and optimizing the polymeric materials comprising the cell [7]. A few prominent examples of this work are: the PCE of acceptors with an innovative design was predicted using machine learning (ML) in [8]; the efficiency of P3HT-based organic solar cells was predicted using an ML statistical data fit in [9]; ML models were trained for the estimation of key parameters such as energy level, UV/visible absorption maxima in solution and film states, and the PCE of PBT7-Th-based solar cells in [10].

Various methods based on elements with unique geometric shapes have made it possible to increase the absorption of a device, such as using concentric mirrors, micro-lenses, optical fibers, and V-shaped cell structures [11,12,13,14,15,16]. Further, implementing physical mechanisms of anti-reflection effects, optical confinement, and increased optical path length (OPL) within the absorber layer can also improve the optical absorption and, therefore, the PCE [17,18]. One technique to increase the OPL is by random scattering using a rough surface, which traps the light within the active medium [17,19,20], resulting in an absorption improvement factor with an upper limit of $4n^{2}$ (the Yablonovitch limit), where *n* is the refractive index of the active layer [21]. Additionally, light scattering can increase absorption via OPL amplification within the absorber layer [22].

Periodically patterned (grating) nanostructures can function as conventional optical elements, such as waveguides, mirrors, and anti-reflection coatings (ARCs), to mention a few, while being much thinner than standard solutions. The integration of such structures can lead to substantially surpassing the conventional absorption limit when optical modes exhibit deep-subwavelength-scale field confinement, opening new avenues for highly efficient next-generation solar cells [17,19,20]. Recent years have witnessed a growing interest in merging grating nanostructures into organic solar cells to improve their performance [23]. These integration approaches utilize the subtle optical effects of the nanostructures beyond their anti-reflection capabilities [17,24,25,26]. Using intelligent choices of materials and the design of the cell’s grating-embedded geometry enables management and manipulation of the electromagnetic (EM) waves that propagate within the device media, thus improving the PCE of the solar cell.

Many complex materials for donors and acceptors have been introduced over the years; these new materials have improved optoelectronic characteristics, charge generation, and charge transport [3]. Currently, the next challenge is to find new ways to improve OSC efficiency and overcome the absorption barrier [27]. Many recent studies have tested the integration of gratings into OSC structures [28,29,30,31]. One of the common solutions is an ARC in the form of a grating layer [28,32]. However, other mechanisms related to the integration of gratings into OSCs can improve OSC efficiency [27,32]. An example of such mechanisms is surface plasmon polariton (SPP) excitation by phase matching [29,30,33], which is based on confining the EM field on top of the grating ridges, within the grating grooves, or both, and thus improving the light trapping in the photo-active layer. It is important to highlight that integrating gratings into OSCs can also provide a broader absorption spectrum and improve angular performance [29,32,33].

By proper design, it has been proven possible to reduce the optical losses via reflection and transmission and to spatially confine the impinging light in the desired regions inside the solar cell device [22,34,35] (e.g., in the absorber to maximize the absorptance and to control the spectral bandwidth to overlap the AM 1.5 solar) [22,34,35]. In addition, the existence of a SPP in the back metal gratings can increase the confinement properties, contributing to the absorption and the short-circuit current density [23,36,37,38]. However, the strong light-polarization sensitivity of solar cells embedded with one-dimensional (1D) gratings has a severe disadvantage for some applications [34,39]. Despite the shortcomings of 1D structures, Li K. et al. found simple grating lines that can perform as well as advanced light-trapping designs [34]. However, in two-dimensional (2D) periodic gratings, such sensitivity becomes negligible, and they are suitable for light trapping improvement design [40,41].

In this study, we examine an organic three-dimensional (3D) solar cell structure embedding two 2D grating nanostructures that are enclosed within the organic semiconductor layer. We used a standard organic solar cell as a case study to test the potential improvement afforded by integrating cuboid and pyramid-grating structures. Our objective was to enhance the optical absorption and the short-circuit current density in order to achieve higher efficiency than that of a standard smooth layered structure. We outline and analyze the optimal designs that achieve the above objective and also show superior absorption over a wide range of azimuthal and polar incidence angles.

## 2. Materials and Methods

### 2.1. Modeling and Simulations

The design and optimization of the proposed organic solar cells, the derivation of their absorption spectra and EM fields, and the simulation of the short-circuit currents were carried out with dedicated rigorous coupled-wave analysis (RCWA) software based on an improved and adapted in-house version. This code and its graphical user interface (GUI) operate in the MATLAB (MathWorks^®^) environment. Integration of this code with a trial-and-error automated multi-start optimization routine [42,43,44] enabled us to perform a real-time optimal computer-aided design of grating multilayer structures.

The optimal design aims to maximize an array of A(λ) samples, for which the sampling is taken from a predefined wavelength vector $\bar{\lambda }$ within the desired wavelength range. Since the objective here is to achieve a broad absorptance spectrum A(λ), the optimization algorithm continues to “wisely” choose values until it succeeds in attaining $A(\bar{\lambda })$ higher than a predefined value or reaches the predefined maximum number of iterations [45,46].

**Figure 1:**
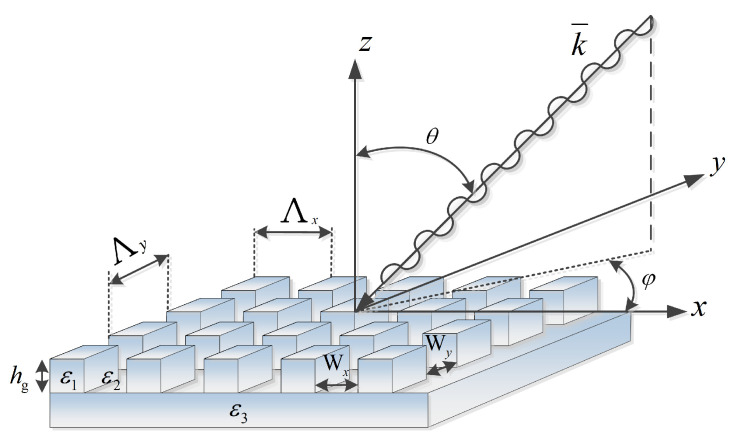
A schematic of a basic structure that comprises a 3D-cuboid-grating layer. The notations on the figure: $\Lambda_{x;y}$, W${}_{x;y}$, $h_{g}$, $\varepsilon_{1}$, $\varepsilon_{2}$, $\varepsilon_{3}$, θ, and φ, are described in the text.

The variable parameters in the optimization process were the grating pitch, Λ, groove width, $W_{x,y}$, and etch depths, $h_{g}$, while keeping the thicknesses of the active layer and smooth layers fixed; parameters are shown in Figure 1. The P3HT:PCBM active layer thickness was 200 nm, excluding structure Pyramid B, where the active layer thickness was reduced to 74 nm. We selected the initial grating pitch Λ values to be equal to the central wavelength (550 nm) of the inspected spectrum range and the initial etch depth $h_{g}$ values to be equal to a quarter of this wavelength. The initial values for the groove width, $W_{x,y}$, were half the grating pitch length.

### 2.2. In-House Simulation Tool

We developed a simulation tool based on the physical-mathematical development of the Maxwell and Helmholtz equations in matrix form for 3D gratings [47]. The mathematical model is based on the RCWA method with improved Fourier–Floquet factorization rules and the scattering matrix propagation algorithm. We examined the far-field properties of the reflectance and transmittance spectra, the EM field spatial profile within the structure, the Poynting vector components, and the absorptance.

Figure 1 shows a schematic of a basic structure that comprises a 3D-cuboid-grating layer, where $\Lambda_{x;y}$, W${}_{x;y}$ are the lateral periods and spacing, respectively, and $h_{g}$ is the grating layer thickness, where $\varepsilon_{1}$ and $\varepsilon_{2}$ are the grating periodic permittivities. This 3D-cuboid-grating is placed on a smooth layer with permittivity $\varepsilon_{3}$. The angles θ and φ are the polar and azimuthal incident angles.

The general solution of the Helmholtz equations in matrix form contains fourth-order tensors (4D matrices) with elements that depend on the Fourier–Floquet properties of the dielectric constant and the conical mounting characteristics. Using the S-Scattering-transformation, boundary conditions for the EM fields, and a recursive process, we obtained the equations for the matrix-tensor transmission and reflection amplitude coefficients, $\eta^{\left(p\right)}$ and $\xi^{\left(p\right)}$, respectively, and for the EM fields within the structure. The simulation tool includes a code developed on the MATLAB platform to solve the final matrix equations. Furthermore, we developed a GUI to simulate and design complex structures, significantly reducing the simulation time. We obtained the near- and far-field EM radiation behavior, which includes reflection and transmission, absorption, spatial fields, and the Poynting vector.

### 2.3. Typical and Grating Organic Solar Cells

**Figure 2:**
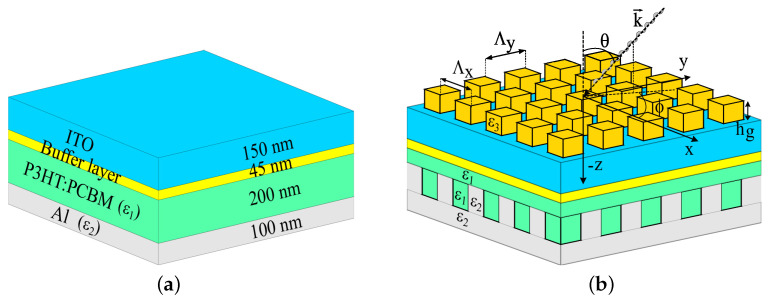
(**a**) The typical organic solar cell structure (the reference structure) consists of smooth layers. (**b**) Structure C: based on the reference organic solar cell structure with front and back grating layers.

**Figure 3:**
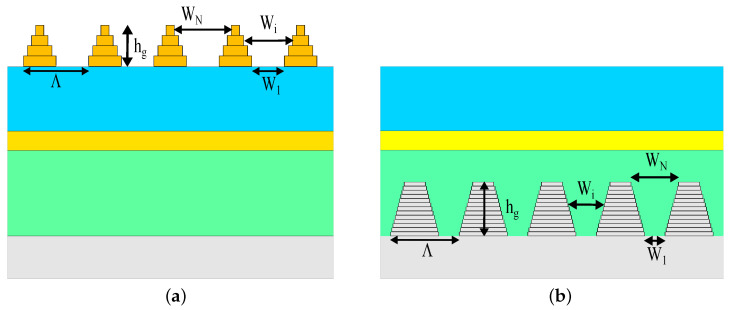
(**a**) Pyramid A structure: a Front 3D-PMMA pyramid-grating layer is placed on the reference solar cell structure. (**b**) Pyramid B structure: a back 3D pyramid-grating layer is integrated at the absorber–aluminum interface of the reference solar cell structure.

We examined six organic solar cell structures—a standard solar cell and five solar cells with integrated gratings. The typical solar cell (Figure 2a) consists of a smooth layer structure based on parameters taken from [48], and it is used as a reference solar cell. The layers are made of an indium tin oxide (ITO) transparent anode (150 nm), PEDOT:PSS (45 nm), an organic absorber of the P3HT:PCBM blend (200 nm), and a non-transparent cathode of an aluminum (Al) layer (100 nm). The five solar cell structures consist of 3D grating layers integrated into the typical cell, as described below:

- A top 3D-PMMA cuboid-grating layer is placed on the reference solar cell structure, which we refer to as structure A;
- A back (rear) 3D-cuboid-grating is integrated at the absorber–aluminum interface of the reference solar cell structure, which we refer to as structure B;
- Front and back cuboid-grating layers with the same periods but with different shifts and duty cycles, which we refer to as structure C configuration (Figure 2b);
- A top 3D-PMMA pyramid-grating layer is placed on the reference structure, which we refer to as structure Pyramid A (Figure 3a) and consists of 4 PMMA/Air cuboid-grating layers forming a truncated square-based pyramid;
- A back 3D pyramid-grating layer is integrated at the absorber–aluminum interface of the reference structure, which consists of 13 P3HT:PCBM/Al cuboid-grating layers forming a truncated square-based pyramid that we refer to as structure Pyramid B (Figure 3b).

We first analyzed the solar cell structures at normal incidence under unpolarized plane-wave irradiation. The investigated spectral range was 400–700 nm, correlated with the absorption spectra of the P3HT:PCBM absorber (active) layer. In this study, we investigated the short-circuit current density of the device, calculated with the help of Equation (1) [18]:

(1) $$J_{\mathrm{SC},\mathrm{total}}=\eta_{\mathrm{int}}\int_{\lambda_{1}}^{\lambda_{2}}\underset{J_{\mathrm{SC}}\left(\lambda \right)}{\underbrace{\frac{q}{hc}\lambda A\left(\lambda \right)\Phi_{AM1.5}\left(\lambda \right)}}d\lambda$$

where λ is the wavelength, A(λ) is the absorptance in the active layer, $\Phi_{\mathrm{AM}1.5}\left(\lambda \right)$ represents the AM 1.5 solar photon flux, and $\eta_{\mathrm{int}}$ is the internal quantum efficiency (we assume $\eta_{\mathrm{int}}=1$). The absorptance A(λ) values were received from the simulation tool, as described in the previous section.

The solar cell efficiency is proportional to the maximum output power from the solar cell, $P_{\max}=V_{\mathrm{OC}}\cdot J_{\mathrm{SC}}\cdot FF$, where FF is the fill factor and $V_{\mathrm{OC}}$ is the open-circuit voltage; this is because the $V_{\mathrm{OC}}$ is affected mainly by the energy levels of the materials [25,49]. We can express the FF as a function of $V_{\mathrm{OC}}$ for all the P3HT:PCBM structures we examined [25,49]. We used the data presented by Rahman et al. [49] on the $V_{\mathrm{OC}}$ for the P3HT:PCBM active material and the mathematical expressions for obtaining the FF. The efficiency will thus be linearly proportional to the short-circuit current density ($J_{\mathrm{SC}}$).

The next step, after we analyzed the structures under unpolarized plane-wave irradiation, was to test the structures’ tolerance to a range of incident angles. We tested the structures’ tolerance to a range of polar (θ) and azimuthal (φ) incident angles. We analyzed the spectral-angular absorptance A(λ,θ,φ) over the relevant λ range. The results of this analysis are presented in Section 3.4.

## 3. Results and Discussion

**Figure 4:**
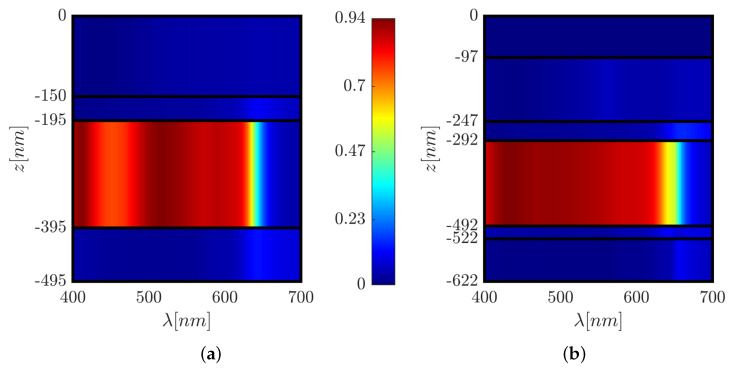
Color map of the spectral absorptance in each layer. (**a**) Reference structure. (**b**) Solar cell structure C.

**Figure 5:**
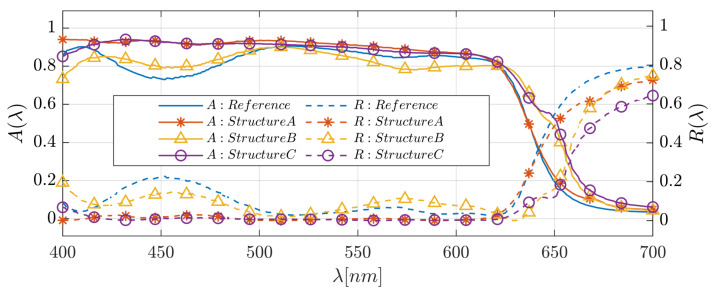
The spectral absorptance in the active layer (full lines) and the reflection efficiency (dashed lines) for the various cells.

**Figure 6:**
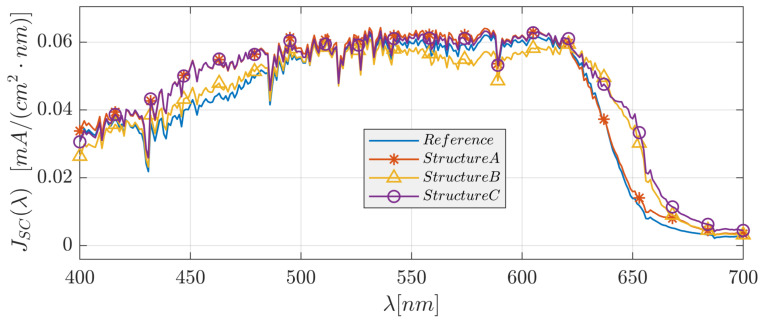
The short-circuit current density spectra of the reference solar cell structure and solar cell structures A, B, and C.

We examined the optical and physical properties of the six solar cell structures described above, including the spatial and spectral distribution of the absorption within the layers and the spectral short-circuit current density and reflection. Figure 4 shows the absorptance color map in each layer as a function of wavelength for the reference solar cell structure and solar cell structure C. In Figure 5, we present the spectral absorption in the active layer and the reflection efficiency for the reference solar cell structure and structures A, B, and C; Figure 6 shows these structures’ short-circuit current density spectrum. The front grating in solar cell designs A, C, and Pyramid A are used to couple the incident light with large propagation angles (θ) into higher-order propagation modes and enhance the conversion efficiency [50].

### 3.1. A Top 3D-PMMA Cuboid-Grating Layer–Structure A

In solar cell structure A, we added a 3D-PMMA grating layer on top of the ITO layer of the reference structure. Optimizing the structural parameters for normal incidence resulted in the following values: Λ=400 nm, grating height $h_{g}=120$ nm, *x*-axis duty cycle = 0.35 ($DC_{x}=\left(\Lambda -W_{x}\right)/\Lambda$), and *y*-axis duty cycle = 0.9 ($DC_{y}=\left(\Lambda -W_{y}\right)/\Lambda$). A color map of the spatial absorption of the solar cell reference structure is shown in Figure 4a. The spectral absorption in the absorber layer (*A*(λ)) and the reflection (R(λ)), and the short-circuit current density ($J_{\mathrm{SC}}$(λ )) are shown in Figure 5 and Figure 6, respectively. For both structures (the reference solar cell structure and solar cell structure A), the absorptance and reflectance have a similar bandwidth. However, solar cell structure A exhibits improved and smoothed absorptance and reflectance compared to the reference solar cell structure. Furthermore, adding the grating layer improved the anti-reflection properties. As a result of this anti-reflection improvement, the short-circuit current density increased by 7.47%. It is noted that the absorptance is very low for λ>∼630 nm due to the P3HT:PCBM absorption properties.

### 3.2. A Back 3D-Cuboid-Grating Layer–Structure B

In solar cell structure B, we embedded a 3D-cuboid-grating layer of P3HT:PCBM and Al between the P3HT:PCBM layer and the contact Al layer. The reason for investigating this structure was to observe the effect of the embedded grating on the optical confinement properties in the absorber layer, where the absorber layer can be seen as a confinement waveguide. Matching between “scattered” modes from the grating layer and the guided modes in the waveguide can enhance the light confinement properties and improve the solar cell absorptance. Optimizing the structural parameters resulted in the following values: Λ=365 nm, grating height $h_{g}=30$ nm, $DC_{x}=0.485$, and $DC_{y}=0.930$; the smooth absorber layer thickness = 200 nm. It is apparent from the results that the spectral bandwidth of the absorptance and reflectance increased. However, there are oscillations in the absorptance spectrum (Figure 5), which are barely noticeable in structures A and C. The short-circuit current density increased by 2.86%. The broadening of the bandwidth is due to the appearance of plasmons in the back-Al grating. These plasmons may affect the confinement properties in the absorber layer above the Al gratings.

### 3.3. Top and Back 3D-Cuboid-Grating Layers–Structure C Configuration

**Figure 7:**
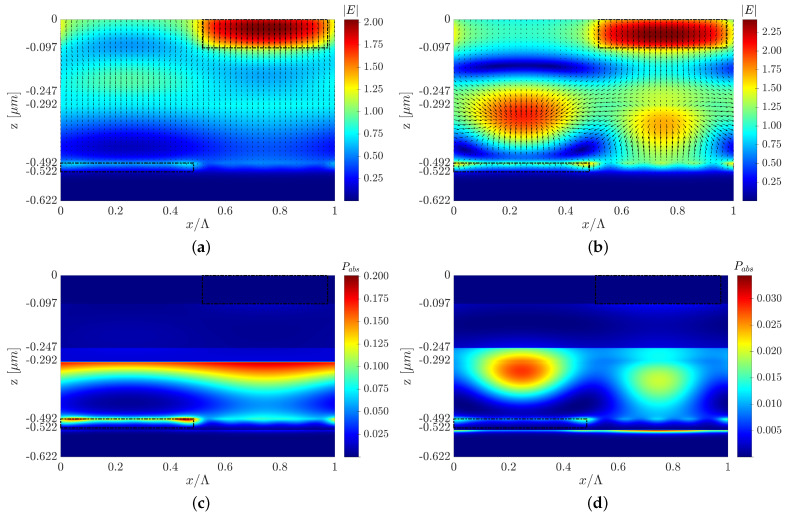
Electric field intensity and quiver plot of the Poynting vector of structure C at (**a**) λ=600 nm and (**b**) λ=650 nm, and the normalized absorbed power profile color map of structure C at (**c**) λ=600 nm and (**d**) λ=650 nm, where the y cross-section is at the center of the P3HT:PCBM grating mesa.

**Figure 8:**
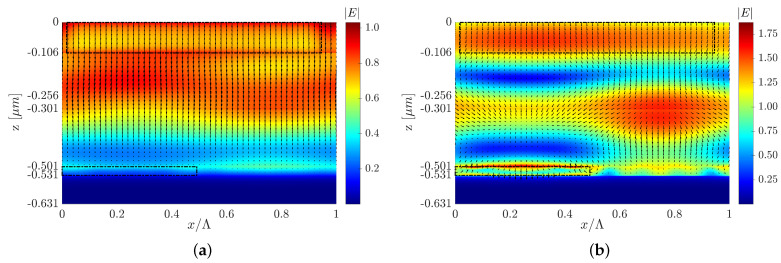
Electric field intensity and quiver plot of the Poynting vector of structure C${}_{1}$ at (**a**) λ=600 nm and (**b**) λ=650 nm, where the y cross-section is at the center of the P3HT:PCBM grating mesa.

This section presents two optimized structures with a type C configuration with top and back 3D-cuboid-grating layers, structures C and C${}_{1}$. We also present another optimized structure design to show that changes in the grating layer’s parameters can affect the field’s behavior, the Poynting vector, and the mechanism behind the solar cell absorptance. However, even though the EM fields and Poynting vector have different behaviors, it is possible to obtain a similar improvement in the cell’s efficiency. Each design induces different EM field and Poynting vector distributions, as shown in Figure 7a,b and Figure 8. However, both designs display a similar increase in the short-circuit current density compared to the reference structure.Figure 7c,d show only structure C’s normalized absorbed power profile color map, but in both designs (C and C${}_{1}$), the high absorbed power is obtained in the P3HT:PCBM active layer. Figure 8b shows an example of the spatial Poynting vector and its vortex in the back-Al grating of structure C${}_{1}$. An enhanced electric field intensity adjacent to and above the Al grating can be seen—note the red area in Figure 8b, which represents the high electric field intensity above the grating mesa. The normalized absorbed power ($P_{\mathrm{abs}}$) is obtained from the absorption per unit volume due to material absorption calculated from the divergence of the Poynting vector [51]:

(2) $$P_{\mathrm{absorption}}=-0.5\cdot Re\left\{\nabla \cdot \vec{S}\right\}=-0.5\cdot \omega \cdot {\left|E\left(\omega \right)\right|}^{2}\cdot Im\left\{\varepsilon \left(\omega \right)\right\}$$

where $\vec{S}$ is the Poynting vector, ω is the angular frequency, ${\left|E\left(\omega \right)\right|}^{2}$ is the squared amplitude of the electric field, and ε(ω) is the material’s permittivity. We received the normalized absorbed power from the calculated $P_{\mathrm{absorption}}$, where $P_{\mathrm{abs}}=P_{\mathrm{absorption}}/\max(P_{\mathrm{absorption}}$). Figure 7c,d presents the absorbed power profile color map at λ=600 nm and λ=650 nm, respectively, but the same can be shown for any other wavelength in the solar cell’s spectral range. We can see that the absorbed power is lower at λ=650nm than that obtained at λ=600nm, which is expected from the absorptance spectra in Figure 5; however, for both wavelengths, the absorption power is concentrated in the P3HT:PCBM active layer. We assumed that the absorbed power in the Al metal regions does not contribute to the solar cell power. Similarly, it is possible to design different grating layers that will result in similar improvements in solar cell efficiency for the other structure configurations (structure A, structure B, Pyramid A, and Pyramid B).

These structures are based on a combination of both the A and B structure configurations, as shown in Figure 2b, where the optimized grating layer structural parameters of structure C are: $\Lambda_{\mathrm{top}}=365$ nm, $h_{g,\mathrm{top}}=97$ nm, $DC_{x,\mathrm{top}}=0.457$, and $DC_{y,\mathrm{top}}=0.930$, $\Lambda_{\mathrm{back}}=365$ nm, $h_{g,\mathrm{back}}$=30 nm, $DC_{x,\mathrm{back}}=0.485$, and $DC_{y,\mathrm{back}}=0.930$, and those of structure C${}_{1}$ are: $\Lambda_{\mathrm{top}}=378$ nm, $h_{g,\mathrm{top}}=106$ nm, $DC_{x,\mathrm{top}}=0.930$, and $DC_{y,\mathrm{top}}=0.444$, $\Lambda_{\mathrm{back}}=378$ nm, $h_{g,\mathrm{back}}$=30 nm, $DC_{x,\mathrm{back}}=0.491$, and $DC_{y,\mathrm{back}}=0.575$. Structure C’s top grating is shifted relative to the bottom grating by 0.52·Λ in the *x*-direction and 0.02·Λ in the *y*-direction, and structure C${}_{1}$’s top grating is shifted relative to the bottom grating by 0.02·Λ in the *x*-direction and 0.47·Λ in the *y*-direction. All the other structural parameters were unchanged. The spectral bandwidths of the absorptance and reflectance increased significantly, whereas the short-circuit current density increased by 10.88% compared to the reference structure (Figure 5 and Figure 6). The impact on the physical properties—the anti-reflection properties due to the top grating (structure A) and the enhanced confinement and plasmon properties due to the back grating (structure B)—was also evident in structure C. In addition, the two grating layers can function as resonant cavity mirrors that may increase the absorptance.

### 3.4. Pyramid 3D Grating Structures

The truncated 3D pyramid structures consist of cuboid grating layers. We studied two types of 3D pyramid structures: (a) Pyramid A, constructed from 4 PMMA/Air cuboid-grating layers on top of the reference structure (Figure 3a); (b) Pyramid B, constructed from 13 Al/P3HT:PCBM cuboid-grating layers on the back of the reference structure at the P3HT:PCBM absorber–aluminum interface (Figure 3b). To obtain a maximal short-circuit current density, we found the optimal number of gratings layers. The best results were obtained for a top pyramid-grating with 4 cuboid-grating layers (Pyramid A) and a back pyramid-grating with 13 cuboid-grating layers (Pyramid B). In each pyramid configuration, the cuboid-grating layers are of equal thickness.

The obtained structural parameters from the optimization process are (a) Pyramid A, with a top 3D pyramid-grating: grating pitch Λ=400 nm, grating height $h_{g}=169$ nm, $W_{x,y,(i)}=\left(19.2+95.2\cdot (i-1)\right)$ nm, where *i* is the cuboid-grating layer number and is an integer between 1 and 4; (b) Pyramid B, with a back 3D pyramid-grating: grating pitch Λ=184, grating height $h_{g}=126$ nm, $W_{x,y,(i)}=\left(50+8.85\cdot (i-1)\right)$ nm, where *i* is the cuboid-grating layer number and is an integer between 1 and 13.

**Figure 9:**
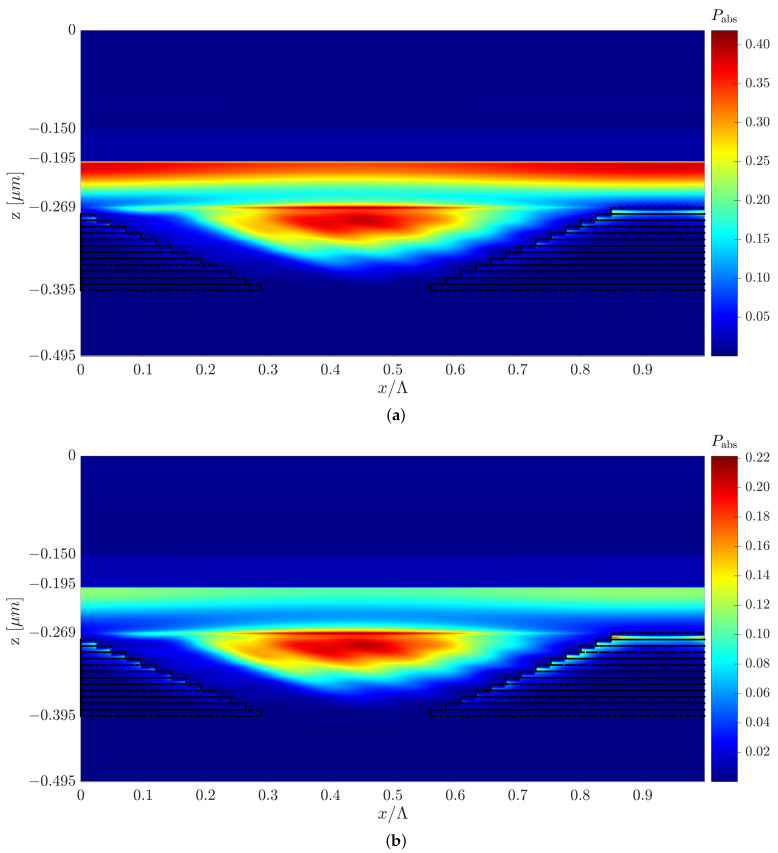
Normalized absorbed power profile color map of Pyramid B at (**a**) λ=500 nm and (**b**) λ=600 nm, where the y cross-section is at the center of the P3HT:PCBM grating mesa.

**Figure 10:**
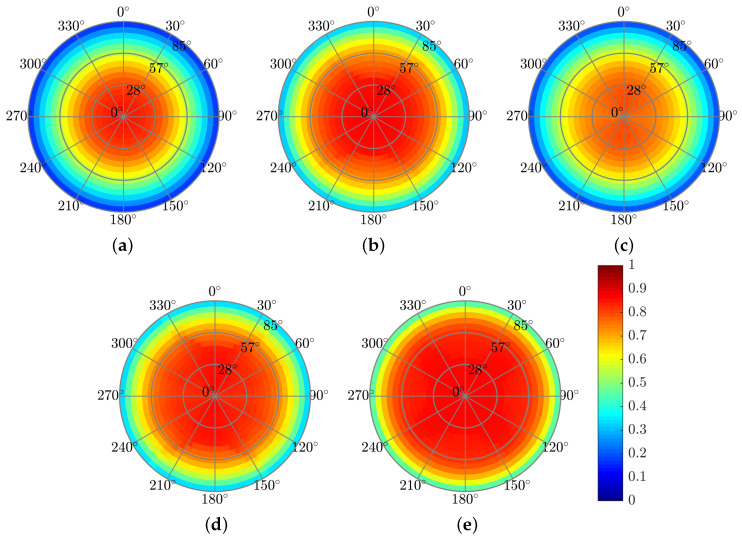
Absorptance color maps showing dependence on the polar (θ${}^{\circ }$-radius) and azimuthal (ϕ${}^{\circ }$-angle) incident angles at λ = 600 nm. (**a**) Reference. (**b**) Structure A. (**c**) Structure B. (**d**) Structure C. (**e**) Pyramid A.

**Figure 11:**
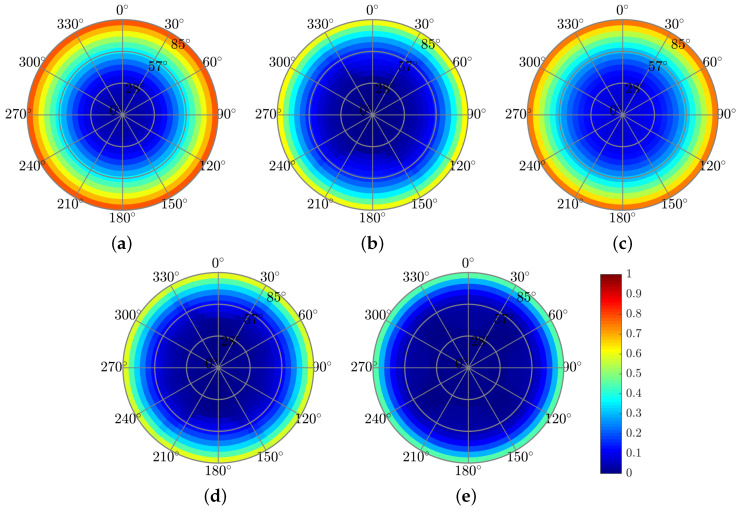
Reflection color maps showing dependence on the polar (θ${}^{\circ }$-radius) and azimuthal (ϕ${}^{\circ }$-angle) incident angles at λ = 600 nm. (**a**) Reference solar cell. (**b**) Structure A. (**c**) Structure B. (**d**) Structure C. (**e**) Pyramid A structure.

The effects of the pyramid-grating on the absorptance, reflectance, and short-circuit current density are similar to those for the rectangular grating for normal incidence. The advantage of the back-embedded pyramid-grating layer is that it can function as a V-groove light trapping concentrator or act as a nano black-body layer, increasing the absorption for a wide range of azimuthal and polar incidence angles. The improvement may be due to the electromagnetic coupling mechanism in the truncated square-based layers in the top pyramids, where each layer acts as a resonator, and the plasmonic resonators in the truncated square-based layers are in the bottom pyramids [52]. Figure 9 presents the color map of the absorbed power profile. The advantage of using the top pyramid-grating layer is the improvement in the anti-reflection effect for a wide range of incidence angles (θ,φ), as shown in Figure 10 and Figure 11, which present the color map of the absorptance depending on both the azimuthal and the polar angles. In general, the absorptance of all the solar cell structures is much more sensitive to changes in the polar angle (θ) than in the azimuthal angle (φ). Figure 10 shows a decrease in the absorptance along the circled color map radius (θ) and its relative stability along the circle’s circumference (φ). The solar cell structures with the top grating layer (solar cell structures A, C, and Pyramid A) showed lower dependency on the polar angle, whereas Pyramid A presented the best results. This improvement can be explained by claiming that the pyramid acts as a black body cavity in which the incident radiation is scattered within the cavity resulting in lower reflectivity for wide incident angles [53].

A summary of the optimal normalized short-circuit current densities of the various grating structures studied is shown in Table 1. The normalization is relative to the short-circuit current density of the reference structure based on parameters taken from [48]. We calculated efficiency PCE${}_{a}$ only considering the absorption in the smooth active layer, while efficiency PCE${}_{t}$ also considers the absorption in the absorbing material of the back grating layer.

## 4. Conclusions

This study aimed to improve the absorption performance of an organic solar cell and to increase its absorption for a wide range of azimuthal and polar incidence angles. We focused on enhancing the short-circuit current density, leading to higher efficiency than achieved with a reference smooth layered structure. We outlined and analyzed optimal designs that displayed improved absorption, short-circuit current density and efficiency compared with the standard reference structure.

We examined the effect of rectangular and pyramid 3D gratings embedded at selected locations in an organic solar cell structure. We used an in-house tool based on the RCWA method developed on the MATLAB platform for the simulations and optimizations of the proposed solar cell structure designs. With the help of this simulation and optimization software, we examined the spectral properties of the absorption, reflection, and short-circuit current density and their dependence on the incident angle for the optimal design structures. All these properties improved due to the addition of the grating layers. For example, in one of the proposed configurations, the short-circuit current density increased by 10.88%, excluding the contribution of the absorbing material in the back grating layer that increases the short-circuit current density by an additional 7.64% to a total of 18.53%.

Adding the top grating layers improved the anti-reflection properties. Adding the back-embedded grating structure improved the light confinement properties due to matching the “diffracted” modes and the confined modes in the active layer and the appearance of SPPs. The advantage of using pyramid structures compared to rectangular gratings lies in improving the anti-reflection effect. In addition, the embedded pyramid-grating structure can function as a light-trapping concentrator, increasing the absorption for a wide range of azimuthal and polar incidence angles. Moreover, Pyramid B, including the contribution of the absorbing material in the back pyramid-grating, achieved the highest increase in short-circuit current density with an additional 24.04% to a total of 26.95%. In this study, we used a standard organic solar cell as a reference structure to test the potential improvement afforded by integrating cuboid and pyramid-grating structures. After analyzing the results, we claim that careful design and integration of grating structures can improve various organic solar cell structures.

## Figures and Tables

**Table 1:** Optimized grating parameters, normalized short-circuit current density, and power conversion efficiency (PCE) of the studied structures. The normalization is relative to the short-circuit current density of the reference structure. PCE${}_{a}$ considers only the absorption in the smooth active layer; PCE${}_{t}$ considers the absorption in the smooth active layer and that of the absorbing material in the back grating layer.

Structure Type	$\Lambda_{x,y}$ (nm)	$h_{g_{\mathrm{top}}}$ (nm)	$h_{g_{\mathrm{back}}}$ (nm)	$J_{{\mathrm{SC}}_{\mathrm{Normalized}}}$	PCE${}_{a}$ (%)	PCE${}_{t}$ (%)
Reference	-	-	-	1.0000	6.53	-
A	400	120	-	1.0747	7.01	-
B	365	-	30	1.0286	6.71	7.20
C	365	97	30	1.1088	7.24	7.74
Pyramid A	400	169	-	1.0510	6.86	-
Pyramid B	184	-	126	1.0298	6.72	8.29

## Data Availability

Not applicable.

## Abbreviations

The following abbreviations are used in this manuscript:

PCE	power conversion efficiency
OPL	optical path length
EM	electromagnetic
RCWA	rigorous coupled wave analysis
ITO	indium tin oxide
P3HT	poly(3-hexylthiophene)
PCBM	phenyl C61-butyric acid methyl ester
